# Apolipoprotein E Gene Polymorphism, Glycated Hemoglobin, and Peripheral Arterial Disease Risk in Chinese Type 2 Diabetic Patients

**DOI:** 10.1155/2020/6040525

**Published:** 2020-03-07

**Authors:** Yujing Hu, Tinghuan Ling, Min Yu, Yang Bai, Tongbao Feng, Ping Zhang, Yan Wang

**Affiliations:** Department of Clinical Laboratory, The Affiliated Changzhou No.2 People's Hospital of Nanjing Medical University, Changzhou, China

## Abstract

**Background:**

The apolipoprotein E (ApoE) gene polymorphism has been found to influence plasma lipid concentration, and its correlation with peripheral arterial disease (PAD) has been investigated. However, it is unclear whether ApoE is associated with PAD in Chinese type 2 diabetes mellitus (T2DM) patients. Therefore, our study is aimed at investigating the relationship between the ApoE gene polymorphism and PAD in Chinese T2DM patients.

**Methods:**

A total of 192 T2DM patients were divided into two groups: T2DM and T2DM with PAD. The clinical and biochemical parameters were obtained. Polymerase chain reaction was used to identify the genotypes of ApoE. The multivariable logistic regression analysis was used to identify the possible risk factor for PAD.

**Results:**

There were no significant differences in the genotype and allele frequencies of ApoE between the T2DM and T2DM with PAD groups. However, the T2DM with PAD group tended to have more *ε*4/*ε*4/

**Conclusions:**

These results demonstrated that there was no evidence of a relationship between ApoE and PAD.

## 1. Introduction

Diabetic patients with peripheral arterial disease (PAD) are at higher risk of increased morbidity compared to healthy people [1]. PAD is a disease in which atherosclerotic plaques cause arterial obstruction and reduce blood flow [2] and can increase the risk of limb and cardiovascular events [3, 4].

Previous studies have shown that the prevalence of PAD increases with age, smoking, and diabetes [5, 6]. Recently, it is well accepted that genetic factors are associated with PAD [7], and apolipoprotein E (ApoE) is one such potential genetic factor [8, 9]. The mechanism of PAD related to ApoE might be attributable to lipid profiles. ApoE plays an important role in lipid metabolism and is responsible for altering circulating levels of cholesterol [10, 11]. There are three alleles for ApoE (*ε*2, *ε*3, and *ε*4), and these alleles in turn form six different ApoE genotypes (*ε*2/*ε*2, *ε*3/*ε*2, *ε*3/*ε*3, *ε*4/*ε*2, *ε*4/*ε*3, and *ε*4/*ε*4) [12]. Generally, the *ε*4 allele better corresponds with higher cholesterol levels and vascular disease risk than the *ε*2 allele and the *ε*3 allele [13, 14]. Multiple studies have investigated the association between ApoE and PAD; however, the results are inconsistent. According to a metastudy, there was no relation between ApoE and PAD in elderly Japanese-American men [8]. However, a recent study found that the *ε*2*ε*2 genotype was associated with an increased risk for PAD in patients at high risk for cardiovascular disease [9].

To date, no studies have evaluated the association between ApoE and PAD in Chinese type 2 diabetes mellitus (T2DM) patients. Therefore, this study is aimed at investigating the possible effect of ApoE on PAD in Chinese T2DM patients.

## 2. Materials and Methods

### 2.1. Patients and Samples

The study protocol had been approved by the Ethics Committee of Changzhou No.2 People's Hospital. Written or verbal consent forms were obtained from each patient, and the motive of the study was clearly explained to them. A total of 192 T2DM patients were recruited from Changzhou No.2 People's Hospital from January 2017 to October 2017. T2DM was defined as fasting plasma glucose ≥ 7.0 mmol/L or 2 h oral glucose tolerance test ≥ 11.1 mmol/L according to the criteria of the World Health Organization [15]. 122 patients were men and 70 were women with ages ranging from 21 to 87 years. These patients were divided into two groups: T2DM and T2DM with PAD. T2DM without PAD included 71 subjects who were free of PAD symptom, type 1 diabetes mellitus, hepatic disease, and other metabolic diseases. T2DM with PAD (T2DM+PAD) included 121 subjects who were confirmed PAD by the angioplasty or ankle-brachial pressure index (ABI). Exclusion criteria included those having type 1 diabetes mellitus, hepatic disease, and other metabolic diseases. Questionnaires were used to collect the information of sex, age, medical and family history of diabetes, smoking habits, and hypertension. Smokers were defined as those who had once smoked even if they were no longer smokers. Hypertension was defined as blood pressure above 140/90 mmHg or taking antihypertensive drugs. Body height and weight were measured, and body mass index (BMI) was calculated as weight (kg) divided by height in meters squared.

### 2.2. ApoE Genetic Analysis

2 mL of fresh blood sample was taken from each patient and collected in EDTA-containing tubes. Genomic DNA was extracted from peripheral blood using the DNA extraction kit (QIAGEN, Germany) and amplified by polymerase chain reaction (PCR). The primers for this reaction were sense 5′ AACAACTGACCCCGGTGGCG 3′ and antisense 5′ ATGGCGCTGAGGCCGCGCTC 3′ [16]. PCR began with an initial denaturation at 94°C (5 min), then 30 cycles at 94°C (45 s), 65°C (45 s), and 72°C (45 s), followed by a final extension at 72°C (5 min). ApoE genotypes were performed using TaqMan® allelic discrimination assays (Applied Biosystems, Foster City, CA, US). For detection of ApoE c.334T>C (ref SNP ID: rs429358), we used assay ID: C_3084973_20, and for c.472C>T (ref SNP ID: rs7421), we used assay ID: C_904973_10.

### 2.3. Measurements of Peripheral Arterial Disease

Briefly, the ankle-brachial index (ABI) was derived from the calculation of the ratio of the ankle systolic blood pressure (SBP) divided by the arm SBP [17]. The ankle and arm blood pressures were determined by the ABI-form device (VaSera VS-1000, Japan). The diagnostic criterion for PAD was an ABI < 0.9 [18, 19]. The participant was also classified as having PAD if there was at least one stenosis of more than 50% in a major artery or one of their branches during the angioplasty examination.

### 2.4. Biochemical Measurements

Blood samples were collected from the patients after 10-12 h overnight fast and then immediately determined within 1 h. Total cholesterol (TC), triglycerides (TG), high-density lipoprotein (HDL), and low-density lipoprotein (LDL) were measured by an automated analyzer (Cobas 8000, Germany). Glycated hemoglobin (HbA1c) values were determined by the high-performance liquid chromatography method (Tosoh G8, Japan).

### 2.5. Statistical Analysis

All statistical analyses were performed with SPSS 16.0 (SPSS Inc., Chicago, USA). Data from normally distributed parameters are presented as the mean ± SD. Independent sample *t*-tests were performed to find any differences in the age, disease duration, BMI, HbA1c, TC, HDL, LDL, and TG between the T2DM and T2DM with PAD groups. Categorical data, such as sex, hypertension, family history of diabetes, and smoking habits, were analyzed between the two groups by the chi-squared test or Fisher's exact test. The frequency differences of the ApoE allele and genotype between groups were tested by chi-squared tests or Fisher's exact test. The adjusted odds ratio (OR) with 95% confidence interval (CI) was used to determine the independent risk factor for PAD by multivariate logistic regression analysis after adjusting for age, sex, BMI, TC, TG, HDL, LDL, HbA1c, smoking habits, hypertension, disease duration, family history of diabetes, and ApoE. Statistical significance was defined at *P* < 0.05.

## 3. Results

### 3.1. The Demographic and Biochemical Parameters of the Study Population

Initially, a total of 240 Chinese T2DM patients participated in the present study. 48 subjects were excluded due to uncompleted questionnaires, unsuccessful genotyping, or both. Finally, 192 participants were included in the sample analysis. The demographic and biochemical data of the patients are shown in Table 1. Among the T2DM with PAD patients, 39 (32.2%) cases were female and 63 (52.1%) subjects were smokers. Information on their medical and family history indicated that 75 (62.0%) patients had hypertension and 49 (40.5%) had a family history of diabetes. Significant differences were observed in age, disease duration, smoking, and hypertension between T2DM patients with and without PAD. Other clinical data, such as sex, family history of diabetes, TC, TG, HDL, LDL, and HbA1c, did not show a statistically significant difference between the two groups. Also, the T2DM patients with PAD had similar BMI levels as the T2DM patients.

### 3.2. Genotypes and Allele Frequency of ApoE

The distributions of the ApoE genotypes and allele frequency are shown in Tables 2 and 3, respectively. The genotype frequencies of all patients were in Hardy-Weinberg equilibrium. Allele frequencies were estimated by gene counting. There were no significant differences in the genotype and allele frequencies of ApoE between T2DM alone and T2DM with PAD patients. However, the T2DM with PAD group tended to have more *ε*4/*ε*3 genotypes (21.5% vs. 11.3%) than the T2DM group. Also, the *ε*4 allele was more frequent, and the *ε*2 allele was less frequent in the T2DM with PAD group, but the differences were not statistically significant.

### 3.3. Association of ApoE Alleles with Lipid Profiles

The relationship between ApoE alleles and plasma lipid levels is shown in Tables 4 and 5. Patients in each group (T2DM and T2DM with PAD) were divided into *ε*3/*ε*3 carriers (*ε*3/*ε*3), *ε*4 carriers (*ε*3/*ε*4 and *ε*4/*ε*4), and *ε*2 carriers (*ε*2/*ε*3 and *ε*2/*ε*2). Two participants who had the *ε*2/*ε*4 genotype were excluded from this analysis because of their opposing effect on lipid metabolism. There were no significant differences in plasma lipid levels among three ApoE allelic groups. Additionally, we did not find significant differences in the lipid profile between *ε*3/*ε*3 carriers, *ε*4 carriers, and *ε*2 carriers in either group.

### 3.4. Association of Clinical Parameters with PAD Risk

The multivariate logistic regression analysis was used to analyze the clinical factors correlated with PAD, all subjects were considered as a whole, and the presence of PAD was considered as a dependent variable (1 = PAD and 0 = non − PAD); sex (1 = male and 2 = female), hypertension (1 = hypertension and 0 = nonhypertension), smoking (1 = smoker and 0 = nonsmoker), family history of diabetes (1 = family history of diabetes and 0 = no family history of diabetes), ApoE (1 = ApoE2 (*ε*2/*ε*2 and *ε*3/*ε*2), 2 = ApoE3 (*ε*3/*ε*3 and *ε*4/*ε*2), and 3 = ApoE4 (*ε*4/*ε*3 and *ε*4/*ε*4)), BMI, TC, TG, HDL, LDL, HbA1c, and disease duration were considered as covariates. The results are shown in Table 6. We found that smoking, age, disease duration, TG, LDL, and HbA1c were significant predictors of the presence of PAD (*P* = 0.006, OR = 2.710, 95%CI = 1.325‐5.541 (smoking); *P* = 0.000, OR = 1.067, 95%CI = 1.032‐1.105 (age); *P* = 0.036, OR = 1.070, 95%CI = 1.005‐1.141 (disease duration); *P* = 0.013, OR = 1.593, 95%CI = 1.103‐2.301 (TG); *P* = 0.001, OR = 2.073, 95%CI = 1.338‐3.213 (LDL); and *P* = 0.029, OR = 0.830, 95%CI = 0.703‐0.981 (HbA1c)).

## 4. Discussion

In our study, there was no relationship between ApoE and PAD in Chinese T2DM patients. However, the T2DM with PAD group tended to have more *ε*4/*ε*3 genotypes (21.5% vs. 11.3%) than the T2DM group. Further studies are needed to explore the potential role of ApoE in PAD in Chinese T2DM patients.

The association of ApoE and the lipid profile remains controversial. The *ε*4 allele is associated with a high level of serum TC and LDL in the Chinese population. To demonstrate that the *ε*4 allele has a great influence on the lipid levels, we analyzed the plasma lipid levels among three ApoE allelic groups and between *ε*3/*ε*3 carriers, *ε*4 carriers, and *ε*2 carriers in both groups, respectively. Although *ε*4 carriers had higher levels of LDL than *ε*3/*ε*3 and *ε*2 carriers, the differences were not statistically significant. This might be due to the differences in genetic background and dietary restrictions and lipid-lowering aggressive treatment in the patients.

In this study, PAD refers to arterial occlusive disease of the lower and upper limbs [20], and its occurrence and development relate to many factors such as hyperglycemia, abnormal lipid metabolism, inflammatory variables, ethnicity, and genotype [7]. ApoE has been identified as an important candidate gene in PAD. Therefore, many studies have been conducted to define the relationship between ApoE and PAD. The SMART study analyzed the ApoE relationship with PAD in a wide sample of patients and found that the *ε*2/*ε*2 genotype seemed to be related to both mild and severe PAD [9]. In a southern Italy study, a higher incidence of total peripheral revascularizations (defined as carotid plus lower limb revascularizations) in *ε*4 carriers was observed [14]. In a Japanese-American large elderly sample, no relationship was found between ApoE and PAD, but the *ε*4/*ε*3 group with newly diagnosed and prevalent diabetes had a significantly higher prevalence of PAD than other diabetic subjects among ever-smokers [8]. In our research, the T2DM with PAD patients tended to have more of genotype *ε*4/*ε*3 than the T2DM patients, but this did not reach statistical significance. This lack of significance might be due to the relatively small number of patients and the influence of ethnic differences. It is clear that the frequencies of ApoE allele and genotype vary between different populations [10, 21]. Therefore, further larger studies are needed to explore the potential role of ApoE in PAD.

The traditional risk factors (age [22], smoking [23, 24], and dyslipidemia [25–27]) have been studied widely in relation to PAD, which was consistent with our observations. The reason for this is that smoking can enhance oxidative stress and oxidative stress can increase vascular inflammation, leading to PAD [28]. The potential explanation for dyslipidemia is that it can cause increased inflammation, monocyte activation, and endothelial dysfunction [29]. A significant age difference was found between the two groups which indicated that the increase in age could possibly lead to higher chances of developing PAD [30].

Also, although the level of HbA1c is lower in the T2DM with PAD patients than the T2DM patient, we found that HbA1c might be a risk factor for PAD in Chinese T2DM patients. The lower level of HbA1c in the T2DM with PAD group might be due to better DM treatment in the higher risk population. HbA1c is the product of nonenzymatic glycation reaction of hemoglobin and glucose in the blood, and the advanced glycation end products (AGEs) are produced by glucose, protein, and lipid through nonenzymatic glycosylation reactions [31], so HbA1c is related to AGEs. Moreover, AGEs are associated with PAD. First, they might promote the generation of reactive oxygen species and inactivate nitric oxide (NO), resulting in the increased formation of the toxic by-product of NO, peroxynitrite [32, 33]. Moreover, AGEs are critical for inhibiting endothelial cell-derived NO production by the suppression of endothelial NO synthase expression, leading to vasodilation, inflammatory reactions, platelet activation, and aggregation [34, 35]. Finally, they might lead to the migration of foam cells, accelerating the progression of atherosclerosis [36].

In terms of limitations, our study population is limited to T2DM patients. As such, our results might not be applicable to healthier individuals. In addition, this study is limited to the Chinese sample size. Thus, the genetic association found in this study might not be generalized to other ethnic populations. Finally, we did not collect data on drug therapy, and we plan to collect the above data in our further study.

## 5. Conclusions

In summary, our study found that there was no relationship between ApoE and PAD. Moreover, smoking, age, disease duration, TG, and LDL can aggravate the progression of both diseases. Furthermore, T2DM patients identified to carry high-risk factors should be treated aggressively to prevent the progression of PAD.

## Figures and Tables

**Table 1:** The demographic and biochemical parameters of T2DM patients compared with T2DM+PAD patients.

Parameters	T2DM (*n* = 71)	T2DM+PAD (*n* = 121)	*P*
Age (years)	52.93 ± 13.21	61.05 ± 10.68	0.001^∗^
Sex (female) (*n* (%))	31 (43.7%)	39 (32.2%)	0.112
Disease duration (years)	5.59 ± 5.86	9.09 ± 6.75	0.001^∗^
Hypertension (*n* (%))	28 (39.4%)	75 (62.0%)	0.002^∗^
Smoking (*n* (%))	26 (36.6%)	63 (52.1%)	0.038^∗^
Family DM history (*n* (%))	31 (43.7%)	49 (40.5%)	0.668
BMI (kg/m^2^)	25.22 ± 3.31	25.12 ± 3.11	0.832
TG (mmol/L)	1.94 ± 1.06	2.15 ± 1.06	0.181
TC (mmol/L)	4.35 ± 1.00	4.47 ± 1.04	0.406
HDL (mmol/L)	1.13 ± 0.33	1.08 ± 0.25	0.267
LDL (mmol/L)	2.12 ± 0.78	2.33 ± 0.97	0.092
HbA1c (%)	9.75 ± 2.41	9.18 ± 2.03	0.084

^∗^ *P* < 0.05. T2DM: type 2 diabetes mellitus; PAD: peripheral arterial disease; BMI: body mass index; TG: triglycerides; TC: total cholesterol; HDL: high-density lipoprotein; LDL: low-density lipoprotein; HbA1c: glycated hemoglobin.

**Table 2:** The distribution of ApoE genotypes in T2DM patients with and without PAD.

ApoE genotypes	T2DM (*n* = 71)	T2DM+PAD (*n* = 121)	*P*
*ε*2/*ε*2 (*n* (%))	1 (1.4%)	1 (0.8%)	1.000
*ε*3/*ε*2 (*n* (%))	12 (16.9%)	14 (11.6%)	0.297
*ε*3/*ε*3 (*n* (%))	47 (66.2%)	78 (64.5%)	0.808
*ε*4/*ε*2 (*n* (%))	1 (1.4%)	1 (0.8%)	1.000
*ε*4/*ε*3 (*n* (%))	8 (11.3%)	26 (21.5%)	0.073
*ε*4/*ε*4 (*n* (%))	2 (2.8%)	1 (0.8%)	0.556

T2DM: type 2 diabetes mellitus; PAD: peripheral arterial disease.

**Table 3:** ApoE allele distribution in T2DM patients with and without PAD.

ApoE alleles	T2DM (*n* = 142)	T2DM+PAD (*n* = 242)	*P*
*ε*2 (*n* (%))	15 (10.5%)	17 (7.0%)	0.226
*ε*3 (*n* (%))	114 (80.3%)	196 (81.0%)	0.865
*ε*4 (*n* (%))	13 (9.2%)	29 (12.0%)	0.391

T2DM: type 2 diabetes mellitus; PAD: peripheral arterial disease.

**Table 4:** The association of lipid profile parameters and ApoE allele in T2DM patients.

Group name	Number	TC	TG	HDL	LDL
A	13	4.29 ± 0.92	2.11 ± 1.17	1.09 ± 0.20	1.93 ± 0.72
B	47	4.39 ± 0.99	1.92 ± 1.07	1.14 ± 0.38	2.12 ± 0.78
C	10	4.17 ± 1.27	1.76 ± 1.02	1.14 ± 0.23	2.35 ± 0.91
LSD-t, *P*	A vs. B	0.759	0.566	0.627	0.437
	A vs. C	0.773	0.442	0.695	0.218
	B vs. C	0.533	0.679	0.971	0.427

T2DM: type 2 diabetes mellitus; PAD: peripheral arterial disease; TG: triglycerides; TC: total cholesterol; HDL: high-density lipoprotein; LDL: low-density lipoprotein; A: *ε*2 carriers (*ε*2/*ε*3 and *ε*2/*ε*2) of T2DM patients; B: *ε*3/*ε*3 carriers (*ε*3/*ε*3) of T2DM patients; C: *ε*4 carriers (*ε*3/*ε*4 and *ε*4/*ε*4) of T2DM patients.

**Table 5:** The association of lipid profile parameters and ApoE allele in T2DM with PAD patients.

Group name	Number	TC	TG	HDL	LDL
A	15	4.65 ± 1.18	2.25 ± 1.35	1.15 ± 0.24	2.21 ± 0.97
B	78	4.38 ± 1.05	2.04 ± 0.97	1.07 ± 0.22	2.36 ± 0.96
C	27	4.64 ± 0.96	2.34 ± 1.12	1.10 ± 0.33	2.38 ± 0.99
LSD-t, *P*	A vs. B	0.375	0.485	0.222	0.580
	A vs. C	0.980	0.790	0.479	0.597
	B vs. C	0.279	0.207	0.600	0.949

T2DM: type 2 diabetes mellitus; PAD: peripheral arterial disease; TG: triglycerides; TC: total cholesterol; HDL: high-density lipoprotein; LDL: low-density lipoprotein; A: *ε*2 carriers (*ε*2/*ε*3 and *ε*2/*ε*2) of T2DM with PAD patients; B: *ε*3/*ε*3 carriers (*ε*3/*ε*3) of T2DM with PAD patients; C: *ε*4 carriers (*ε*3/*ε*4 and *ε*4/*ε*4) of T2DM with PAD patients.

**Table 6:** Multivariate logistic regression analysis of the factors correlated with PAD.

Parameters	OR	95% CI	*P*
Smoking	2.710	1.325-5.541	0.006^∗^
Age	1.067	1.032-1.105	0.001^∗^
Disease duration	1.070	1.005-1.141	0.036^∗^
Sex	0.507	0.166-1.552	0.234
Hypertension	1.696	0.767-3.751	0.192
Family history of diabetes	1.088	0.518-2.284	0.823
TG	1.593	1.103-2.301	0.013^∗^
LDL	2.073	1.338-3.213	0.001^∗^
HbA1c	0.830	0.703-0.981	0.029^∗^
BMI	0.945	0.835-1.069	0.366
TC	0.978	0.558-1.714	0.937
HDL	0.594	0.121-2.914	0.521
Apolipoprotein E genotype	1.467	0.793-2.712	0.222

^∗^ *P* < 0.05. Analysis of the data was done using multivariate logistic regression analysis (adopted factors: sex, age, disease duration, smoking, hypertension, family history of diabetes, BMI, TC, TG, HDL, LDL, HbA1c, and ApoE). PAD: peripheral arterial disease; OR: odds ratio; CI: confidence interval; BMI: body mass index; TG: triglycerides; TC: total cholesterol; HDL: high-density lipoprotein; LDL: low-density lipoprotein; HbA1c: glycated hemoglobin; ApoE: apolipoprotein E.

## Data Availability

Not applicable. The conclusions of the manuscript are based on relevant datasets, which are available in the manuscript.
